# Generation of gene-edited rats by delivery of CRISPR/Cas9 protein and donor DNA into intact zygotes using electroporation

**DOI:** 10.1038/s41598-017-16328-y

**Published:** 2017-11-29

**Authors:** Séverine Remy, Vanessa Chenouard, Laurent Tesson, Claire Usal, Séverine Ménoret, Lucas Brusselle, Jean-Marie Heslan, Tuan Huan Nguyen, Jeremy Bellien, Jean Merot, Anne De Cian, Carine Giovannangeli, Jean-Paul Concordet, Ignacio Anegon

**Affiliations:** 1 Centre de Recherche en Transplantation et Immunologie UMR1064, INSERM, Université de Nantes, Nantes, France; 2 Institut de Transplantation Urologie Néphrologie (ITUN), CHU Nantes, Nantes, France; 3 Platform Transgenic Rats and ImmunoPhenomics, INSERM UMR 1064-CRTI, F44093 Nantes, France; 4 Platform GenoCellEdit, INSERM UMR 1064-CRTI, F44093 Nantes, France; 5 INSERM U1096, F76031 Rouen, France; 6 Institut du thorax, INSERM UMR 1087, CNRS UMR 6291, F44007 Nantes, France; 7 INSERM U565, CNRS UMR7196, Museum National d’Histoire Naturelle, F75005 Paris, France

## Abstract

The generation of gene-edited animals using the CRISPRs/Cas9 system is based on microinjection into zygotes which is inefficient, time consuming and demands high technical skills. We report the optimization of an electroporation method for intact rat zygotes using sgRNAs and Cas9 protein in combination or not with ssODNs (~100 nt). This resulted in high frequency of knockouts, between 15 and 50% of analyzed animals. Importantly, using ssODNs as donor template resulted in precise knock-in mutations in 25–100% of analyzed animals, comparable to microinjection. Electroporation of long ssDNA or dsDNA donors successfully used in microinjection in the past did not allow generation of genome-edited animals despite dsDNA visualization within zygotes. Thus, simultaneous electroporation of a large number of intact rat zygotes is a rapid, simple, and efficient method for the generation of a variety of genome-edited rats.

## Introduction

Engineered nucleases, such as zinc-finger nucleases (ZFN), transcription activator-like effector nucleases (TALEN) and more recently clustered regularly interspaced short palindromic repeats (CRISPR)-associated (Cas) system, are powerful tools for targeting any sequence directly in mammalian zygotes, and obtaining genetically modified animals, especially species in which there are no robust embryonic stem cells, such as rats but also farm animals, zebra fish, xenopus, etc^1–5^. These molecular scissors introduce a double-strand break (DSB) at the target locus, which could be repaired by non-homologous end joining (NHEJ), introducing insertions or deletions (indels) that if are not multiple of 3 nt result in frame shift of the coding sequence with generation of premature stop codons, degradation of the whole mRNA and as a consequence generation of knockout animals. The other potential repair mechanism in presence of single-stranded DNA (ssODNs) or double-strand DNA (dsDNA) carrying sequences homologous to the target site is the homology-directed repair (HDR) pathway. Both targeted genome editing pathways, NHEJ and HDR, have been performed until now by microinjection of these nucleases and donor DNA directly into every processed zygote. Even if this method is efficient, it requires specific skills, expensive equipment and is time-consuming.

Electroporation is a simple technique that is widely used for introducing macromolecules e.g proteins, DNA, mRNA, etc… into cultured cells, living tissues and more rarely and recently into preimplantation embryos^6–8^. The interest of electroporation is that time is shorter vs. microinjection (for 50 zygotes a few seconds vs. 1 hour, respectively), requires an equipment less expensive than for microinjection and no special technical skills are needed. Nevertheless, the efficiency of electroporation depends on the electroporation parameters (e.g. electric field intensity, pulse length, pulse repeat), the cargo to incorporate, but also on the own properties of the target cell. In the case of one-cell-stage embryos, the presence of the zona pellucida (ZP) makes difficult the introduction of macromolecules. To overcome this physical barrier, the ZP is weakened either by a chemical treatment (Tyrod’s acid) or by application of a strong electric field to create pores. Although the weakening of the ZP allows to better control the electroporation efficiency, many studies have shown that the early developmental potential of zona-free embryos is often compromised, particularly in rats^9^.

Several groups have recently showed that the delivery of ZFN, TALEN or CRISPR-Cas9 components using zygote electroporation enables generation of mice^10–13^ or rats^14,15^ carrying various targeted genetic modifications, with variable efficiencies depending on nucleases and strategies chosen. In several of these studies, electroporation was done in zygotes that were denudated of the ZP^10,11,16^. Different publications showed deliver of CRISPR-Cas9 components into zygotes to generate NHEJ-mediated indel mutations^10–15^, large segment deletions^11,13^, and to introduce HDR-mediated precise nucleotide substitutions^10,11,15^ or short sequence insertions^11,12,16^ using ssODNs. To date, no data showing the introduction of large DNA fragment by delivering engineered nucleases into mouse or rat zygotes through electroporation has been reported in the literature.

In this study, we report the application of electroporation on intact rat zygotes using CRISPR/Cas9 technology to successfully generate gene-inactivating mutations by NHEJ and targeted mutations (indels and short segment insertion using ~100 nt ssDNA) into several selected genomic loci of intact rat zygotes. Insertion of large DNA fragment using superposed short single-stranded oligodeoxynucleotides (ssODN) or long ss or dsDNA were not obtained.

## Results

### Gene editing using CRISPR/Cas9 protein and ssODN templates for HDR

To introduce CRISPR/Cas9 protein and template DNA into intact rat zygotes, we used the Nepa21 electroporator, a 3-step pulses system, which in a first step allows making micro-holes in the ZP and cytoplasmic membrane (“poring pulse”, high voltage and short duration), and in a second and third time allows transferring DNA, mRNA, protein cargos into zygotes cytoplasm (“transfer pulses” low voltage and long duration). The polarity change of the second transfer pulses increases the transfer efficiency.

To optimize the experimental conditions, we tested a series of 4 “poring pulse” of 50 ms interval with a voltage ranging from 250 V to 300 V and duration of pulses, from 0.5 ms to 2.5 ms The first and second transfer pulses were set to 20 V, 50 ms pulse width, 5 pulses of 50 ms interval. Egg viability *in vitro* and in utero was analyzed for each parameter tested. We found that for a same voltage, a pulse duration of 2.5 ms leads to a significant higher toxicity than a pulse duration of 0.5 ms, with a more pronounced impact at 300 V than 250 V (Supplementary Fig. S2). This toxicity is already observed 1 h after electroporation, but also after overnight culture and mostly after 14d of gestation, suggesting that embryo development is substantially affected (Supplementary Fig. S2).

**Figure 1:**
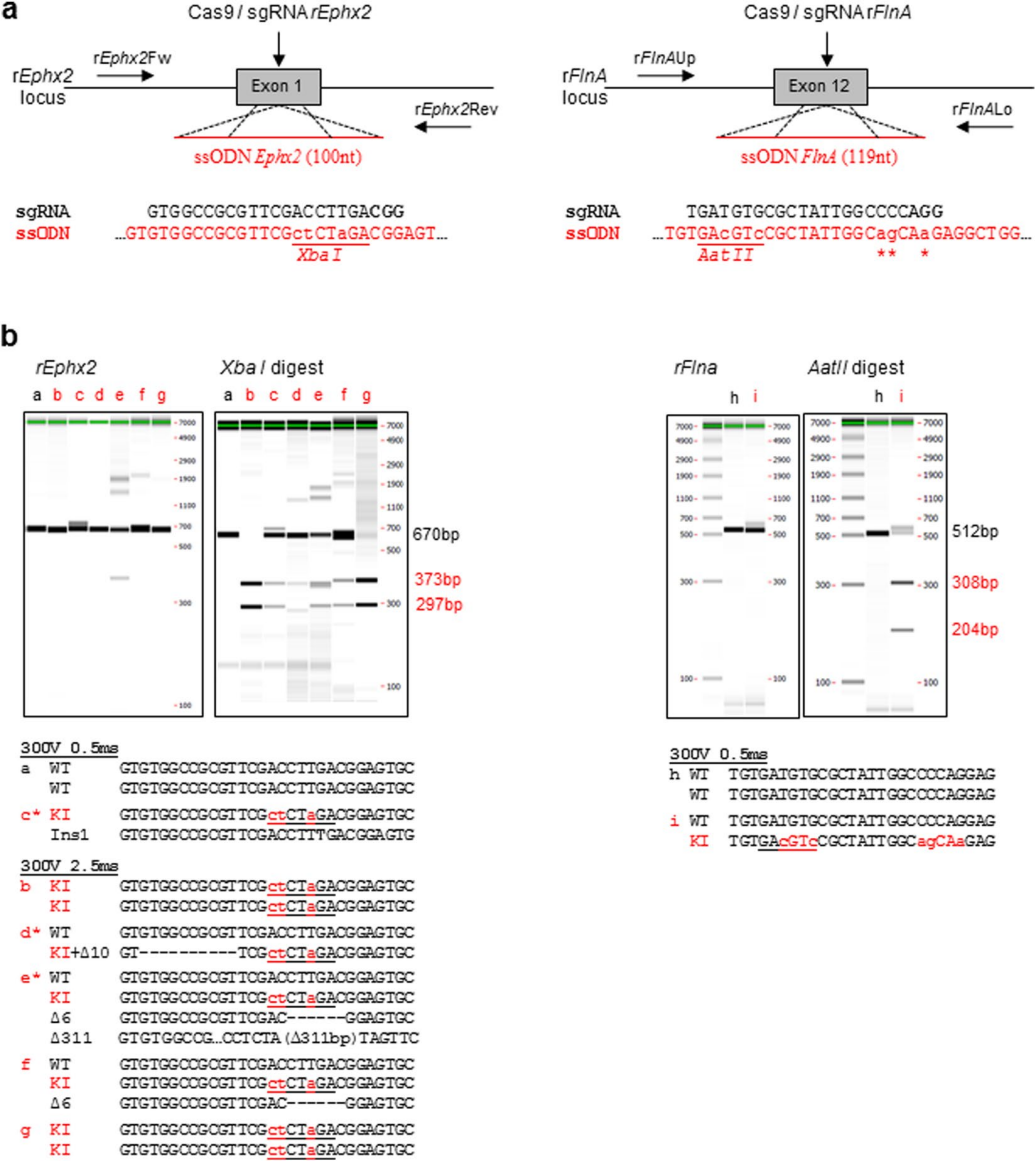
CRISPR/Cas9 mediated HDR strategies using ssODN. (**a**) Schematic illustration of the target sequences + PAM sequence (in bold) into rat *Ephx2* (upper left) and *FlnA* (upper right) loci and the respective ssODN (100 nt and 119 nt in length) designed to insert a *XbaI* or an *AatII* recognition site (underlined in red, small letters mutations introduced into the genome). These mutations prevent further cleavage of the *Ephx2* mutated gene by CRISPR/cas9 and additional mutations were introduced into *FlnA* ssODNs to prevent cleavage of this template (indicated by stars). The position of each primer is indicated on the schematic rat *Ephx2* and *FlnA* loci. (**b**) (upper left) *Ephx2* PCR analysis of a representative panel of 7 rats from the group electroporated with Cas9 (3 µM)/sgRNA *Ephx2* (150 ng/µl)/ssODN (150 ng/µl) using the following poring pulse conditions: 300 V/0.5 ms (#a,#c) and 300 V/2.5 ms (#b,#d,#e,#f,#g). Capillary gels show the *rEphx2* PCR products of the 670 bp expected size and of 373 bp and 297 bp after *XbaI* digestion. All animals with the exception of one (#a) showed *XbaI* digestion and two animals (#b and #g) showed disappearance of the 670 bp PCR product suggesting homozygous KI insertion. (lower left) Sequencing of the subcloned PCR products from positive animals. Deletions are indicated by “Δ”, insertions by “Ins” and knock-in events are indicated by “KI”. *XbaI* recognition sites are underlined in inserted alleles. In four animals (#c,#d,#e,#f) both NHEJ and knock-in events were observed. Two animals are homozygous KI (#b,#g) and one is WT (#a). *Sequence analyses of clones from animals #c, #d and #e are detailed in Table S2. (upper right) *FlnA* PCR analysis of the 2 representative rats from the group electroporated with Cas9 (3 µM) /sgRNA *FlnA* (150 ng/µl) /ssODN (150 ng/µl) using the poring pulse condition 300 V/0.5 ms. Capillary gels show the *FlnA* PCR products of the 512 bp expected size and of 308 bp and 204 bp after *AatII* digestion. One of both animals (i) showed *AatII* digestion suggesting a knock-in event. (lower right) Sequencing of the PCR products from the positive animal. Knock-in event is indicated by “KI”. *AatII* recognition site is underlined in inserted alleles.

To test the efficiency of electroporation to introduce Cas9 protein complexed to sgRNA (single-guide RNA) and ssODN as donor template to modify the genomic loci, we chose to target *Ephx2* and *Filamin A* (*Flna*) (Fig. 1a). Intact rat embryos were electroporated with different concentrations of Cas9/sgRNA ribonucleoprotein complexes and different conditions of poring pulses (Table 1) maintaining constant the transfer pulses. Electroporated embryos were genotyped at E14 (*Ephx2* locus) or after birth (*FlnA* locus) by PCR locus amplification. Sequencing and enzymatic digestion by XbaI for *Ephx2* locus and AatII for *Flna* locus were performed to identify gene edited rats with indels (NHEJ+) and/or newly introduced sequences (KI+) (Fig. 1b).

**Table 1:** CRISPR/Cas9 protein-mediated genome editing with ssODN matrix.

Target locus	Cas9 (µM)/sgRNA (ng/µl)/ssODN (ng/µl)	Poring pulse conditions: voltage/pulse width	No. of viable embryos/No. of total embryos (%)^a^	Stage of analysis	No. of embryos or pups analyzed/No. of transferred (%)^b^	No. of NHEJ+ (%)^c^	No. of KI+ (%) ^c^	No. of NHEJ+ KI+ (%)^c^	No. of embryos/pups with 3 or more alleles (%)^c^
*Ephx2*	50^*^/10/15	Microinjection	293/373 (79)	pups	82/250 (33)	17 (21)	12 (15)	8 (9.8)	9 (11)
	3/150/150	300 V/0.5 ms	85/85 (100)	E14	20/63 (32)	10 (50)	1 (5)^#^	1 (5)^#^	4 (20)
		300 V/2.5 ms	97/134 (72)	E14	20/87 (23)	12 (60)	5 (25)^#^	3 (15)^#^	2 (10)
	6/200/200	250 V/0.5 ms	62/70 (89)	E14	5/48 (10)	0 (0)	1 (20)	0 (0)	0 (0)
		250 V/2.5 ms	36/54 (67)	E14	1/36 (3)	0 (0)	0 (0)	0 (0)	/
		300 V/0.5 ms	31/36 (86)	E14	3/31 (10)	1 (33)	0 (0)	0 (0)	0 (0)
		300 V/2.5 ms	14/47 (30)	E14	0/14 (0)	/	/	/	/
*FlnA*	3/100/20	Microinjection	121/144 (84)	pups	21/111 (19)	3 (14)	2 (9.6)	1 (4.8)	2 (9.5)
	3/150/150	300 V/0.5 ms	100/103 (97)	pups	12/100 (12)	1 (8)	1 (8)#	0 (0)	0 (0)
		300 V/2.5 ms	70/119 (59)	pups	1†/100 (1.4)	1† (100)	0 (0)	0 (0)	0 (0)

*Cas9 mRNA (ng/µl). ^a^Calculated 1 h after electroporation. ^b^Calculated from the number of transferred eggs. ^c^Calculated from the number of analyzed embryos or pups. ^†^Stillborn pup. ^#^Sequences detailed in Fig. 1.

For *Ephx2* locus, Cas9 protein (3 µM), sgRNA (150 ng/µL) and ssODN (150 ng/µL) were electroporated into rat zygote under a 300 V poring pulse and 2 different pulse duration (0.5 ms and 2.5 ms). When compared to microinjection of the same reagents, both conditions had a comparable impact on embryo survival 1 h after electroporation (from 72 to 100% versus 79% using microinjection) and on embryonic development in utero (from 23 to 32% versus 33% using microinjection) (Table 1). At 300 V / 0.5 ms, 10 out of 20-analysed embryos carried NHEJ-mediated mutations and 1 out 20 carried HDR-mediated KI sequences. Increasing the pulse duration to 2.5 ms maintained or slightly improved NHEJ (12 out of 20) and improved KI frequencies (5 out of 20) (Table 1). Sequences analysis of the subcloned PCR products revealed that among KI rats, 2 of them were homozygous KI (#b, #g) and 4 others (#c, #d, #e, #f) were heterozygous and also carrying indels (Fig. 1b left; Supplementary Table S2). Sequence analyses of clones revealed that animal #c carried one type of indels (Ins 1 bp) in 8 clones out of 11 and the knock-in *XbaI* site in 3 out of 11. The animal #d carried an allele with one type of indels (Del 10 bp) and the knock-in *XbaI* site (1/8 clones), and a WT allele (7/8 clones). The animal #e carried two types of indels (Del 311 bp visible on F0 sequence; Del 6pb in 3/6 clones), the knock-in *XbaI* site (3/6 clones), and a WT sequence (visible on F0 sequence) (Supplementary Table S2). Animals with NHEJ that were not multiple of 3 nt resulted in frame shift of the coding sequence with generation of premature stop codons and KO rats (data not shown).

Compared to microinjection, the tested conditions allowed to obtain higher indel mutations frequencies (2.5 to 3 fold more) and KI rates up to 25% (versus 15% using microinjection).

Increasing the concentration of Cas9 protein (6 µM) as well as sgRNA and ssODN (200 ng/µL each) decreased the viability 1 h after electroporation when using higher pulse with (down to 67 and 30% for 2.5 ms vs. 89 and 86% at 0.5 ms at 250 and 300 V, respectively), decreased in utero development for all conditions (down to 0% at 2.5 ms for both voltages) and did not improve NHEJ or KI frequencies (Table 1).

We then assessed the efficiency of electroporation in another target gene, the *FlnA* locus. We observed a higher toxicity, especially *in utero*, since only a 300 V / 0.5 ms and not 2.5 ms poring pulse generated lived pups. Compared to microinjection of sgRNA targeting the same locus, the birth rate was slightly lower (12% versus 19% using microinjection) (Table 1). For 300 V / 0.5 ms, 8% of pups were NHEJ+ and 8% were KI+, efficiencies slightly reduced compared to those obtained using microinjection (9.6% and 14% respectively) (Table 1). Sequence analyses revealed that the only KI animal was heterozygous KI (#i) (Fig. 1b right). The expected mutation was transmitted to the F1 and F2 generations (rate of 11% and 43%, respectively) (data not shown).

These results showed that the efficiency of electroporation to obtain KO and KI animals using, in intact rat zygotes, CRISPR/Cas9 protein complexes and ssODN as donor DNA was at least comparable to the one obtained by microinjection.

### CRISPR/Cas9 protein-mediated HDR strategies using long dsDNA templates

**Figure 2:**
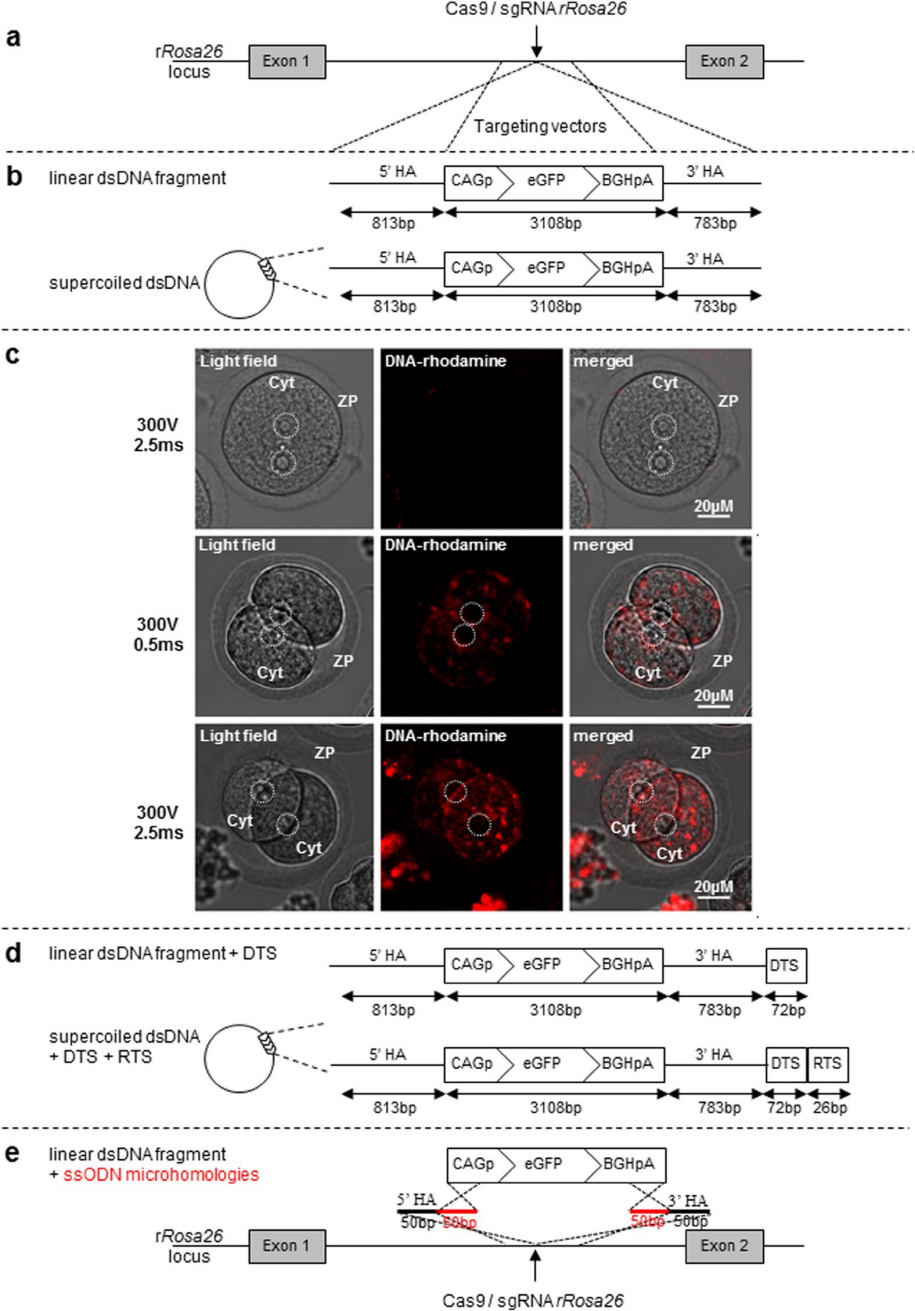
CRISPR/Cas9 mediated HDR strategies using long dsDNA template. (**a**) Schematic representation of the rat *Rosa26* locus with the site of CRISPRs/Cas9 cleavage (arrow). (**b**) The targeting vectors containing a CAG-GFP expression cassette (3108 bp) and two homology arms (5′HA 813 bp and 3′ HA 783 bp) contiguous to the cleavage point. These targeting vectors were either in a linear (excised fragment) or supercoiled form. (**c**) Confocal microscopy images were captured the day after electroporation of rhodamine-labeled linear dsDNA (the same as in **b**) into intact rat zygotes using a 300 v poring pulse and a pulse width adjusted to 0.5 or 2.5 ms and then cultured overnight. (upper) Embryo electroporated with pulse width of 2.5 ms without incorporated DNA. (middle) Embryo electroporated with pulse width of 0.5 ms. (lower) Embryo electroporated with pulse width of 2.5 ms. ZP, zona pellucida; Cyt, cytoplasm. Pronuclei are indicated by dotted circles. (**d**) The same targeting vector as in (**b**) with a DNA targeting sequence (DTS) in a linear form or in a supercoiled form and in this case including *Rosa26* targeted sequences (RTS) to be linearized *in cellulo* by the sgRNA. (**e**) A linear dsDNA containing the same GFP expression cassette as in (**b**) but without *Rosa26* homology arms. Also depicted, the ssODNs containing 50 bp homologies with *Rosa26* sequences at each extremity (in black) and 50 bp homologies with each extremity of the expression cassette (in red).

To assess the efficiency of the method to drive the target integration of a long dsDNA into the rat *Rosa26* locus, we used a donor DNA containing homology arms of 0.8 Kb and a CAG-GFP expression cassette (3.1 Kb) (Fig. 2a), previously shown to obtain HDR in this locus when delivered by microinjection with TALENs mRNA^4^ or with CRISPR/Cas9 as a mRNA or protein complex^17^. This donor DNA was used under a supercoiled plasmid or a linear form after excision of the expression cassette with the homology arms. 100 ng/µl co-administrated with the Cas9-sgRNA (3 µM–150 ng/µl) protein complex into rat zygote using the parameters described above (300 V poring pulse and a 0.5 or 2.5 ms pulse duration). None of the conditions tested allowed a targeted integration of this long dsDNA while we observed up to 100% of NHEJ+ embryos (Fig. 2b) (Table 2). Previous experiments of microinjection of the linear excised DNA donor allowed KI in 1.7% of transferred embryos (Table 2) and no KI with the circular one^17^.

**Table 2:** CRISPR/Cas9 protein-mediated genome editing of *Rosa26* locus with a long dsDNA matrix.

Cas9 (µM)/sgRNA (ng/µl)/dsDNA (ng/µl)	+ssODN Rosa (ng/µl)	Poring pulse conditions: voltage pulse/width	No. of viable embryos/No. of total embryos (%)^a^	Stage of analysis	No. of embryos analyzed/No. of transferred (%)^b^	No. of NHEJ+ (%)^c^	No. of KI+ (%) ^c^
3/100/2^*^	/	Microinjection	121/160 (76)	E14	57/117 (49)	13 (23)	2 (3.5)
3/150/100^*^	/	300 V/0.5 ms	32/37 (86)	E14	6/32 (19)	1 (17)	0 (0)
		300 V/2.5 ms	28/47 (60)	E14	5/28 (18)	0 (0)	0 (0)
3/150/100^**^	/	300 V/0.5 ms	31/31 (100)	E14	19/31 (61)	18 (95)	0 (0)
		300 V/2.5 ms	24/27 (89)	E14	4/24 (17)	4 (100)	0 (0)
3/150/100^***^	/	300 V/0.5 ms	48/56 (86)	E14	5/48 (10)	2 (40)	0 (0)
		300 V/2.5 ms	31/62 (50)	E14	1/31 (3.2)	0 (0)	0 (0)
3/150/100^****^	/	300 V/0.5 ms	46/48 (96)	E14	14/37 (38)	3 (21)	0 (0)
		300 V/2.5 ms	37/50 (74)	E14	2/37 (5.4)	2 (100)	0 (0)
3/150/100^*^	300	300 V/0.5 ms	30/31 (97)	E14	10/30 (33.3)	6 (60)	0 (0)
		300 V/2.5 ms	59/83 (71)	E14	10/51 (20)	4 (40)	0 (0)

*linear excised. **supercoiled. ***linear excised fragment + DTS sequence. ****Supercoiled + DTS sequence + sgRosa. ^a^Calculated 1 h after electroporation. ^b^Calculated from the number of transferred eggs. ^c^Calculated from the number of analyzed embryos or pups.

We then asked whether the larger size of the donor DNA could be an obstacle for the entry by electroporation into the zygote. To this end, we labeled the excised linear DNA with rhodamine using a DNA labeling (covalent binding) and we analyzed by confocal microscopy whether the DNA could be visualized within the embryo after zygote electroporation and culture. We confirmed that DNA was indeed detectable within two-cell stage embryos (Fig. 2c) whereas zygotes that were microinjected with the same labeled DNA did not show positive signal, probably due to the very low quantity delivered DNA (data not shown). Thus, the absence of KI when using long donor DNA as opposed to ssODNs could not be explained by a physical obstacle for the introduction into the zygote by either the ZP or embryo membrane. In contrast, DNA did not seem cross the nuclear barrier since we didn’t detect labeled- DNA into zygote pronuclei (Fig. 2c and data not shown).

To increase the KI efficiency of the method using long DNAs, two new approaches were tested. The first approach consisted to incorporate a 72bp-DNA targeting sequence (DTS) in the delivered plasmid (Fig. 2d) to potentially increase DNA nuclear translocation as it has been previously described^18^. 100 ng/µl of this donor template were co-delivered with the Cas9-sgRNA (3 µM–150 ng/µl) protein complex, either under a linear form (excised fragment) or as a supercoiled plasmid containing the sgRNA *Rosa26* target sequence (RTS) to allow the linearization *in cellulo*. The second, adapted from^19^, used two short 100 nt ssODN to ligate each cut end of genomic DNA with the donor GFP expression cassette via HDR (Fig. 2e). 300 ng/µl of each of the two ssODN were co-delivered with the Cas9-sgRNA (3 µM–150 ng/µl) protein complex and 100 ng/µl of the CAG-GFP cassette, using parameters mentioned above (300 V − 0.5 ms or 2.5 ms).

Even though we obtained high rate of NHEJ reaching 100% in some conditions, none of the two strategies allowed HDR.

### CRISPR/Cas9 protein-mediated genome editing with a long ssDNA matrix

Recent studies have reported a higher KI efficiency by using long ssDNAs (until 1.6 Kb) with short homology arms as repair donor template rather than dsDNAs^19,20^.

**Figure 3:**
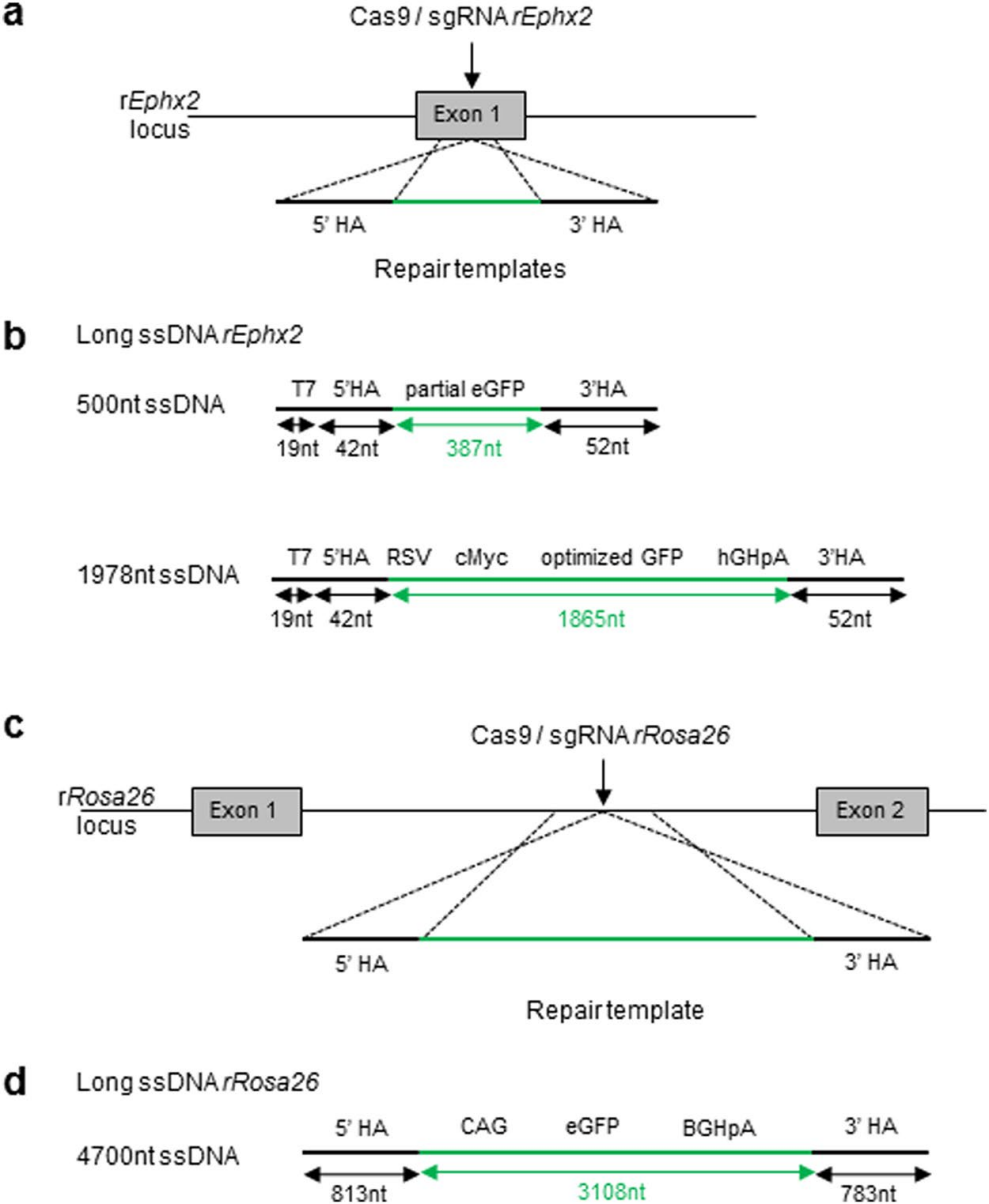
CRISPR/Cas9 mediated HDR strategies using long ssDNA templates. (**a**) Schematic illustration of the rat *Ephx2* locus with the site of CRISPRs/Cas9 cleavage (arrow) and DNA repair templates containing a DNA sequence detailed in (**b**) and two homology arms (5′HA and 3′ HA) contiguous to the cleavage point. (**b**) Strategies tested into *Ephx2* locus include a 500 nt (upper diagram) or a 1978 nt long ssDNA lower diagram), containing respectively a partial GFP sequence (387 nt) or a RSV-GFP-polyA expression cassette (1865 nt) and two homology arms (42 nt in 5′ and 52 nt in 3′). (**c**) Schematic illustration of the rat *Rosa26* locus with the site of CRISPRs/Cas9 action (arrow) and DNA repair templates containing a DNA sequence detailed in (**d**) and two homology arms (5′HA and 3′ HA) contiguous to the cleavage point. (**d**) Strategy tested into *Rosa26* locus includes a 4700 nt long ssDNA, containing a CAG-GFP expression cassette (3108 nt) and two homology arms (5′HA 813 nt and 3′ HA 783 nt).

To test this strategy, we targeted the *Ephx2* and *Rosa26* loci (Fig. 3a and Fig. 3c). For the *Ephx2* locus we *in vitro*-transcribed from PCR product 500 nt and 1978 nt ssDNA containing GFP-encoding sequences flanked of short (42 nt and 52 nt) homology arms as templates (Fig. 3b). For the *Rosa26* locus, we *in vitro*-transcribed from linearized plasmid a 4700 nt ssDNA containing GFP-encoding sequence (3108 nt) flanked by 813 and 783 nt homology arms (Fig. 3d).

20 ng/µl (500 nt and 1978 nt ssDNA), 40 or 60 ng/µl (4700 nt ssDNA) were co-delivered with the Cas9-sgRNA (3 µM-150 ng/µl) protein complex using a poring pulse of 300 V and a pulse duration either of 0.5 ms or 2.5 ms. Genotyping of resulting embryos did not detect KI events in none of the two loci (Table 3).

**Table 3:** CRISPR/Cas9 protein-mediated genome editing with a long ssDNA matrix.

Target locus	Size of ssDNA (nt)	Cas9 (µM)/sgRNA (ng/µl)/ssDNA (ng/µl)	Poring pulse conditions: voltage/pulse width	No. of viable embryos/No. of total embryos (%)^a^	Stage of analysis	No. of embryos analyzed/No. of transferred (%)^b^	No. of NHEJ+ (%)^c^	No. of KI+ (%) ^c^
*Ephx2*	500	3/150/20	300 V/0.5 ms	132/140 (94)	E14	10/91 (11)	0 (0)	0 (0)
			300 V/2.5 ms	88/160 (55)	E14	18/46 (39)	2 (11)	0 (0)
	1978	3/150/20	300 V/0.5 ms	68/70 (97)	E14	29/68 (43)	5 (17)	0 (0)
			300 V/2.5 ms	65/84 (77)	E14	2/36 (5.6)	1 (50)	0 (0)
*Rosa26*	4700	3/150/20	300 V/0.5 ms	35/37 (97)	E14	6/35 (17)	3(50)	0 (0)
			300 V/2.5 ms	25/39 (64)	E14	0/25 (0)	0 (0)	0 (0)
		3/150/40	300 V/0.5 ms	29/30 (97)	E14	4/29 (14)	1 (25)	0 (0)
			300 V/2.5 ms	15/33 (46)	E14	0/15 (0)	0 (0)	0 (0)
		3/150/60	300 V/0.5 ms	35/53 (66)	E14	3/30 (10)	1 (33)	0(0)
			300 V/2.5 ms	30/34 (88)	E14	10/30 (33)	2 (20)	0(0)

^a^Calculated 1 h after electroporation. ^b^Calculated from the number of transferred eggs. ^c^Calculated from the number of analyzed embryos or pups.

### Cas9 protein-mediated genome editing with overlapping ssDNA matrix

**Figure 4:**
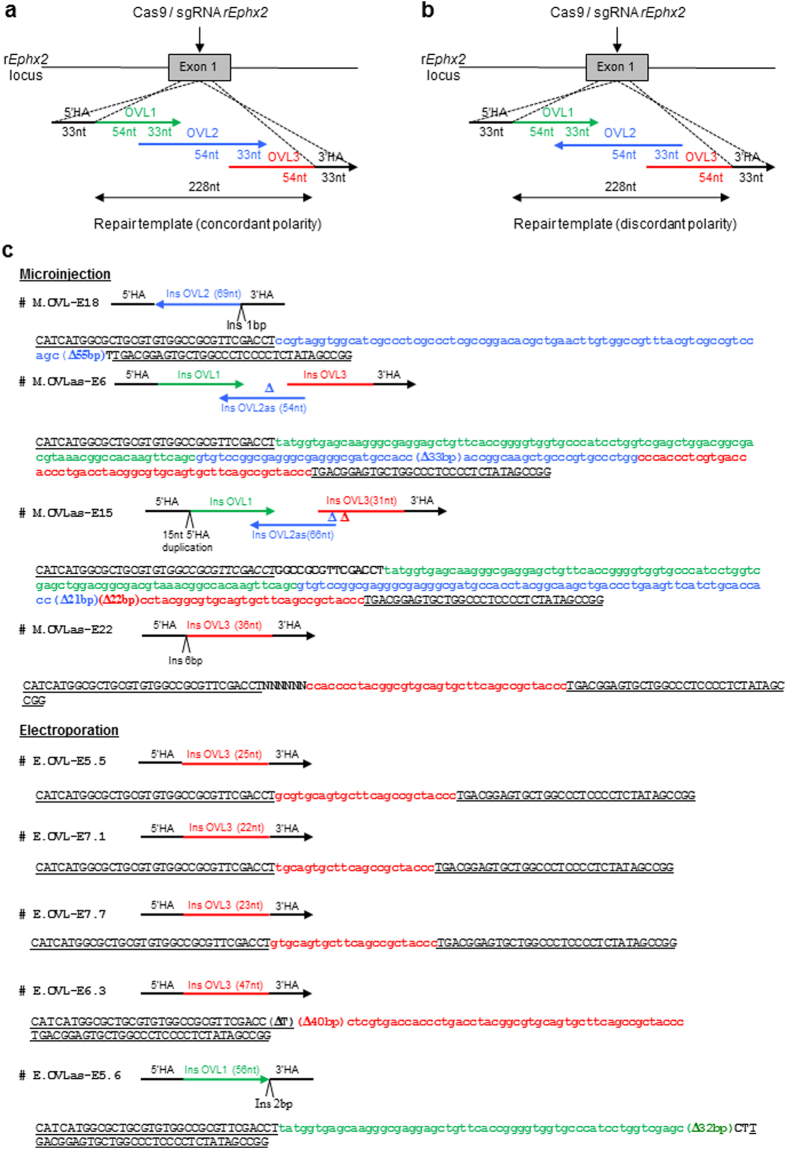
CRISPR/Cas9 mediated HDR strategies using OVL ssODN templates. (**a**) Schematic illustration of the rat *Ephx2* locus with the site of CRISPRs/Cas9 cleavage (arrow) and the 3 overlapping ssODN (OVL1-OVL2-OVL3) of concordant polarity used as DNA repair template. The 3 overlapping ssODN are designed to generate a 228 nt insert at the r*Ephx2* locus. Each ssODN had a 120 nt length. The two external ssODN (OVL1 and OVL3) had a 33 nt overlap with the internal ssODN (OVL2) and a 33 nt HA corresponding to the left or the right side of the DSB. (**b**) Schematic illustration of the rat *Ephx2* locus with the site of CRISPRs/Cas9 action (arrow) and the 3 overlapping ssODN (OVL1-OVL2-OVL3) of discordant polarity used as DNA repair template. The 3 overlapping ssODN are designed to generate a 228 nt insert at the r*Ephx2* locus. Each ssODN had a 120 nt length. The two external ssODN (OVL1 and OVL3) had a 33 nt overlap with the internal ssODN (OVL2) and a 33 nt HA corresponding to the left or the right side of the DSB. (**c**) Sequencing of the subcloned PCR products from embryos for which partial OVL ssODN insertion have been observed. 5′HA and 3′HA are underlined. Deletions are indicated in bold by “Δ”, insertions by “Ins” and in bold uppercase. OVL1, 2 or 3 insertions are indicated respectively in green, blue and red.

We further investigated if the use of overlapping ssODN as repair templates, strategy previously described in *C.elegans* ^21^, could induce long KI sequences into the rat *Ephx2* locus with the similar efficiencies than those observed using a single ~100 nt ssODN, as previously described for ssODNs^11,12,16^ and even using superposed BACs^22^. For that, we synthetized 3 ssODN (OVL1-OVL2-OVL3) of a 120 nt length each, with a 33 nt overlap between the two external ssODN (OVL1 and OVL3) and the internal ssODN (OVL2). The presence of two 33 nt homologous sequences with the rat *Ephx2* locus in 5′ and in 3′ on the OVL1 and OVL3, respectively, and contiguous to the cleavage point would allowed insertion of a 228 nt fragment (Fig. 4a and b). Moreover, we compared the knock-in efficiency in the case or the 3 ssODN have an identical polarity (Fig. 4a) or when the internal ssODN has an opposite polarity to the two others (Fig. 4b)^21^.

Overlapping ssODN (50, 100 or 200 ng/µl each) were co-delivered with the Cas9-sgRNA (3 µM-150 ng/µl) protein complex using a poring pulse of 250 or 300 V and a pulse duration either of 0.5 ms or 2.5 ms. We didn’t obtain any KI events, whatever the tested electroporation parameters, when using ssODN at a concentration of 50 ng/µl each and with a same polarity. In contrast, the cutting rate observed was very efficient ranging from 33% (250 V/2.5 ms) to 100% (300 V/2.5 ms) (Table 4). Increasing the concentration of the overlapping ssODN to 100 ng/µl each allowed to obtained incomplete insertion in 1 animal of 9 (# E.OVL-E5.5) for the condition 250 V/0.5 ms, in 1 of 4 (# E.OVL-E6.3) for the condition 250 V/2.5 ms and in 2 of 16 (# E.OVL-E7.1; # E.OVL-E7.7) for the condition 300 V/0.5 ms (Table 4 and Fig. 4c). We also observed NHEJ rates ranging from 22% to 25% when we applied a 250 V voltage and a 0.5 ms or 2.5 ms pulse length, respectively, and ranging from 25% to 100% for a 300 V voltage and a 0.5 ms or 2.5 ms pulse length, respectively (Table 4). A concentration of ssODNs to 200 ng/µl each did not allow to increase the knock-in efficiencies. As a control, we co-microinjected the 3 overlapping ssODN (20 ng/µl each) with the Cas9-sgRNA (3 µM–150 ng/µl) protein complex into rat one-cell embryo. Genotyping analyses showed a 11% cutting rate, and a partial integration of the repair templates in 1 of the 18 (# M.OVL-E18) analyzed embryos (Table 4 and Fig. 4c).

**Table 4:** CRISPR/Cas9 protein-mediated genome editing with overlapping ssDNA matrix.

Target locus	Repair template	Cas9 (µM)/sgRNA (ng/µl)/ssDNA (ng/µl)	Poring pulse conditions: voltage pulse width	No. of viable embryos/No. of total embryos (%)^a^	Stage of analysis	No. of embryos analyzed/No. of transferred (%)^b^	No. of NHEJ+ (%)^c^	No. of KI+ (%) ^c^	KI+ embryo ID^d^
*Ephx2*	OVL 1.5p-2-3.3p	3/150/3 × 20	Microinjection	78/98 (80)	E14	18/54 (33)	2 (11)	1* (5.6)	M.OVL-E18
		3/150/3 × 50	250 V/0.5 ms	30/30 (100)	E14	4/30 (13)	2 (50)	0 (0)	/
			250 V/2.5 ms	56/58 (97)	E14	3/24 (12.5)	1 (33)	0 (0)	/
			300 V/0.5 ms	27/29 (93)	E14	6/29 (21)	3 (50)	0 (0)	/
			300 V/2.5 ms	24/29 (83)	E14	1/29 (3.4)	1 (100)	0 (0)	/
		3/150/3 × 100	250 V/0.5 ms	36/37 (97)	E14	9/34 (26.5)	2 (22)	1*(11)	E.OVL-E5.5
			250 V/2.5 ms	66/82 (80)	E14	4/66 (6.1)	1 (25)	1*(25)	E.OVL-E6.3
			300 V/0.5 ms	79/82 (96)	E14	16/69 (23)	4 (25)	2* (12.5)	E.OVL-E7.1 E.OVL-E7.7
			300 V/2.5 ms	31/43 (72)	E14	1/31 (3.23)	1 (100)	0 (0)	/
		3/150/3 × 200	300 V/0.5 ms	109/121 (90)	E14	8/70 (11)	6 (75)	0 (0)	/
			300 V/2.5 ms	40/55 (73)	E14	3/40 (7.5)	0 (0)	0 (0)	/
	OVL 1.5p-2 antisens-3.3p	3/150/3 × 20	Microinjection	52/65 (80)	E14	22/52 (42)	6 (27)	3* (14)	M.OVLas-E6 M.OVLas-E15 M.OVLas-E22
		3/150/3 × 50	250 V/0.5 ms	40/42 (95)	E14	10/35 (29)	3 (30)	1* (10)	E.OVLas-E5.6
			250 V/2.5 ms	36/54 (67)	E14	0/36 (0)	/	/	/
			300 V/0.5 ms	43/48 (90)	E14	0/37 (0)	/	/	/
			300 V/2.5 ms	34/60 (57)	E14	0/34 (0)	/	/	/
		3/150/3 × 100	250 V/2.5 ms	85/105 (81)	E14	0/57 (0)	/	/	/
			300 V/0.5 ms	105/116 (91)	E14	8/71 (11)	1 (12.5)	0 (0)	/
			300 V/2.5 ms	123/207 (59)	E14	1/91 (1.1)	0 (0)	0 (0)	/
		3/150/3 × 200	300 V/0.5 ms	52/54 (96)	E14	10/52 (19)	4 (40)	0 (0)	/
			300 V/2.5 ms	36/47 (77)	E14	1/36 (2.8)	0 (0)	0 (0)	/

^a^Calculated 1 h after electroporation. ^b^Calculated from the number of transferred eggs. ^c^Calculated from the number of analyzed embryos or pups. ^d^Sequences of these embryos are in the Fig. 4. *partial insertion.

Concerning the electroporation of the 3 ssODN with a discordant polarity (Fig. 4b), we obtained only 1 positive embryo with an incomplete insertion (# E.OVLas-E5.6) when using ssODN at a concentration of 50 ng/µl each and when series of 250 V/0.5 ms poring pulses were applied. (Table 4 and Fig. 4c). The microinjection of these 3 overlapping ssODN (20 ng/µl each) with the Cas9-sgRNA (3 µM–150 ng/µl) protein complex allowed to obtain a partial integration in 3 embryos on 22 analyzed (# M.OVLas-E6; # M.OVLas-E15; # M.OVLas-E22) (Table 4 and Fig. 4c).

## Discussion

Gene editing in rat embryos has been difficult for decades and has been recently facilitated by the discovery of gene-specific nucleases^23,24^ but microinjection is still a cumbersome and inefficient delivery system. Improvement of delivery systems would allow a more rapid and efficient generation of animal models and analyses of gene function. Several groups have recently used the electroporation method to efficiently deliver Cas9mRNA and sgRNA or Cas9/sgRNA RNP into rat^14,15,25^ or mouse^10–13,15,16^ zygotes. Depending on the electroporation system and the protocol used, a pre-treatment of zygotes with Tyrod’s acid is required to weaken the zona pellucida and so increases delivery efficiency^11,16^. This step not only complicates proceedings but also may affect *in vivo* embryonic development^26,27^.

In this article, we have optimized electroporation protocol with NEPA21 electroporator device, previously described by Kaneko and collaborators^14,15,25^, to efficiently deliver Cas9/sgRNA RNP complexes into intact rat embryos, without compromising *in vivo* embryonic development more than microinjection. Several poring pulses conditions have been tried but 300 V voltage combined to pulse duration of 0.5 ms or 2.5 ms was the optimal condition for which we obtained the highest frequencies of NHEJ and KI events, and an acceptable toxicity level, even if higher at 2.5 ms.

With these parameters, we have successfully generated both indel mutations and HDR-mediated KI using ssODN into 2 different loci, with similar (HDR events) or sometimes higher efficiency (NHEJ events) than using microinjection, depending on either the target locus and/or the sgRNA used as well as the concentrations of Cas9/sgRNA/ssODN. For instance, for *FlnA* locus which was more difficult to edit, even using microinjection, we observed a lower efficiency than that observed for a more permissive locus (*Ephx2*). Two ratios of Cas9/sgRNA/ssODN have been delivered into rat zygotes and the 3/150/150 dose was the best for delivery regardless of target locus. Increasing dose did not improve NHEJ and KI frequencies and considerably affect embryo survival and development.

These results corroborate the previous data obtained in mice and rats and increase the relevance of electroporation combined to the CRISPR technology to generate indel mutations and to introduce HDR-mediated precise nucleotide substitutions^10,11,15^ or short sequence insertions^11,12,16^ using ssODNs. To date, no data showing that is possible to introduce large DNA fragment through electroporation has been reported in the literature. To take up this challenge, we co-electroporated Cas9/sgRNA RNP targeting *Rosa26* locus and a long donor DNA containing a CAG-GFP expression cassette and two homologous sequences previously used to obtain HDR when delivered by microinjection with Cas9 protein^17^. The electroporation parameters tested before and efficient to insert short DNA sequences did not allow a targeted integration of this long donor DNA under a linear or a supercoiled form. As already observed in our previous works based on microinjection of TALEN or CRISPR^4,17^, no integration of the circular DNA form was observed.

Large supercoiled or linear DNA requires larger functional pores for its entry in the cell compared to short ssDNA. Increasing pulse duration or the number of pulses can increase the size of the pores^28^ and so the amount of DNA crossing the ZP and cytoplasmic membranes. Our experiments with rhodamine-labeled long dsDNA showed that the applied poring pulses conditions (300 V/0.5 or 2.5 ms) were efficient for the entry into the cytosol of zygotes. We did not detect DNA into zygote pronuclei suggesting DNA did not cross the nuclear barrier. Several reasons could explain this failure. First, the parameters of electroporation are suboptimal to destabilize the nuclear membrane, preventing DNA entry into the nucleus. The large size of DNA has a reduced electrophoretic mobility in the cytoplasm which may be more pronounced since it has been reported that the higher the voltage and the longer pulse duration are, the higher the size of DNA aggregates is^28^. It is also possible that the microtubule network and the actin filaments, which are involved in the DNA transport in the cytoplasm^29,30^, react to the presence of DNA aggregates and may influence the DNA trafficking within the cell. Finally, because of its limited mobility, DNA molecules are not located at the periphery of nucleus when the membrane is weakened, or are degraded by endonucleases before reaching the nucleus.

To improve the efficiency of the electroporation method to achieve the targeted integration of large dsDNA by HDR, we tested two approaches, the first based on the interaction of DNA with cytosolic proteins, and the second based on ssODN-mediated end joining. The first strategy has shown that DNA binding with some adapter cytosolic proteins, that themselves bind motor proteins, facilitates its traffic and its entry into the nucleus of non-dividing cells^31^. Indeed, the adding of DTS sequence (72 bp enhancer repeat of the SV40 early promoter, a region rich in consensus binding sites for numerous transcription factors) into DNA plasmid can enhance this nuclear import. These sequences form complexes with the nuclear localization signal sequence of certain proteins that in turn bind to importins which facilitate the migration of the complex inside the nucleus^32^. Unfortunately, the incorporation of a DTS sequence in the delivered plasmid (Fig. 2c) to potentially enhance DNA nuclear translocation, did not allow increasing the KI efficiency using long dsDNA template. Concerning the second strategy, it has been recently reported that a DSB DNA repair based on short ssODN used to ligate each cut end of genomic DNA allows integration of large DNA fragment via HDR with higher efficiencies than using donor vector containing long homology arms^19^. We tested this approach by using two short 100 nt ssODN to ligate each cut end of genomic DNA with the donor GFP expression cassette via HDR (Fig. 2e). As well as with the previous approach, this strategy was also unsuccessful.

Taken together, these data suggest that the electroporation method does not allow at this stage to achieve the integration of large dsDNA by HDR into genomic loci of intact rat zygotes. Thus, the size or electric charge of DNA appears to be a limiting factor. Based on these observations, we investigated at what DNA size KI events were not observed anymore. To try to answer this question, we developed two strategies, adapted from previous studies based on the use of long ssDNA^19,20,33^ and overlapping ssODN^21^. For the first one, we used *in vitro* transcribed long ssDNA containing GFP-encoding sequences flanked of short (~50 nt) or long (~800 nt) homology arms, depending of the size of the insert. Unlike the results previously observed with microinjection^19,33^, KI was not observed using electroporation of long ssDNA with short homology arms rather than dsDNA with long homologous sequences. For the second one, we synthetized 3 ssODN of a 120 nt length each, with a 33 nt overlap between the two external ssODN and the internal ssODN. Two 33 nt homology arms are present in 5′ and in 3′ on the OVL1 and OVL3 and corresponding to the right or left side of the DSB (Fig. 4a and b). It has been reported that the repair mechanism is initiated by the OVL3 pairing in 5′ of the DSB followed by DNA synthesis and template switching^21^. We also compared the KI efficiency in the case or the 3 ssODN have an identical polarity (Fig. 4a) or when the internal ssODN has an opposite polarity to the two others (Fig. 4b). Certain conditions of electroporation allowed a partial insertion of only one of the 3 ssODN with efficiencies ranging from 10 to 25%, unlike microinjection for which 2 animals showed an integration of the 3ssODN even if these integrations were not perfect. In contrast to what it has been observed in *C.elegans* ^21^, we observed similar results with overlapping ssODN of same or opposite polarity. Further experiments are needed in terms of polarity and the use of OVL as the opposite strand of the sgRNA.

In conclusion, electroporation is an excellent alternative to microinjection to efficiently deliver Cas9/sgRNA protein complexes and thus perform genome editing in rats with similar (ssODN-mediated HDR) or sometimes higher (NHEJ-mediated indel mutations) success rate but much more easily, rapidly and without requiring operators with training and expertise. Further optimization should lead to introduce large DNA fragment insertion using long ss or ds DNA targeted mutations into genomic loci of intact rat zygote.

## Methods

### Animals

Sprague-Dawley (SD/Crl) rats were the only strain used and were sourced from Charles River (L’Arbresle, France). All the animal care and procedures performed in this study were approved by the Animal Experimentation Ethics Committee of the Pays de la Loire region, France, in accordance with the guidelines from the French National Research Council for the Care and Use of Laboratory Animals (Permit Numbers: CEEA-PdL-2015-692).

### spCas9 and sgRNA preparation

Plasmid pMJ806 was obtained from Addgene (http://www.addgene.org/39312) and modified for the introduction of 3 NLS by ligation independent cloning (Gibson Assembly, New England Biolabs). Expression and purification of *S.pyogenes* Cas9 protein were produced as previously described^17^. sgRNA were designed to target the following sequences: *Rosa26* (GTGTATGAAACTAATCTGTCTGG), *Ephx2* (GTGGCCGCGTTCGACCTTGACGG), *FlnA* (TGATGTGCGCTATTGGCCCCAGG),

sgRNAs were transcribed using the DraI-digested sgRNA expression vectors DR274 as templates and the T7 high yield kit (New England Biolabs). Following transcription, DNase I (New England Biolabs) treatment was carried out and sgRNAs were purified using EZNA microelute RNA Clean-UP column (OMEGA Biotek).

### Preparation of DNA template

The *Ephx2* ssODNs (100 nt), *Ephx2* overlapping ssODNs (120 nt each) and *FlnA* ssODNs (119 nt) were purchased from Eurofins in dry form, dissolved in nuclease free-water at 1 mM and stored at −20 °C until use.

#### Long supercoiled and linear dsDNA donor

Plasmid donor sequences were based on the Brown Norway rat genomic sequence (assembly RGSC_3.4) but were confirmed to match the Charles River Sprague-Dawley rats corresponding sequences. The rat *Rosa26* DNA donor plasmid (7.6 Kb), previously described in detail^4^, contained an expression cassette with the CAG promoter-eGFP cDNA-BGHpA (3108 bp) flanked by two homologous arms sequences (813 bp 5′HA and 783 bp 3′HA) of the rat *Rosa26* locus, contiguous to the site of DNA cleavage (intron 1) by Cas9 (Fig. 2). The plasmid was purified using Endofree plasmid maxi kit (Qiagen, France).

Linear dsDNA donor (4.7 Kb) and linear dsDNA donor without homology arms (3.1 Kb) were produced, respectively, by BstEII or SalI-HindIII restriction of the rat *Rosa26* DNA donor plasmid and purified with kit QIAEX II (Qiagen).

DNA donors were quantified using a NanoDrop-1000 and stored at −20 °C until use.

Each 100 nt ssODN was designed to be homologous to each rROSA cut end (50 nt) and each donor DNA end (50 nt). These ssODNs, named rROSA-CAGp and RBGpA-rROSA, were used to join via HDR the plasmid donor DNA without homology arms and the genomic DNA at the CRISPR-Cas9 cleavage site^19^

rROSA-CAGp

gcattccctctctcctgatcttagaagtccgatgactcatgaaaccagacGTCGACATTGATTATTGACTAGTTATTAATAGTAATCAATTACGGGGTCA

RBGpA-rROSA

TAGCTGTCCCTCTTCTCTTATGAAGATCCCTCGACCTGCAGCCCAAGCTTagattagtttcatacaccacaaatcgaggctgtagctggggcctttaaca

DNA targeting sequence (DTS) was used to improve efficiency of gene delivery. The 72 bp SV40 enhancer has been shown to confer nuclear localization because of its consensus binding sites for transcription factors^34^. DTS sequence was placed at one end of the construct directly following the 3′ HA both in linear or circular construct. The sgROSA target sequence was added to the circular construct in order to linearize the donor DNA in cellulo by the CRISPR-Cas9 system.

The plasmid SK + HAROSACAGGFPpA was linearized by BamHI, dephosphorylated and ligated to SVDTS or SVDTSsgROSA synthetized fragment (Eurofins) digested by BglII-BamHI. Positive clones were tested to check orientation in order to keep the BamHI site at the 3′ end. Linear dsDNA donor with SVDTSsgROSA (4.8 Kb) was produced by BamHI-HindIII restriction and purified with kit QIAEX II (Qiagen).

SK + HAROSACAGGFPpASVDTS:

gatctGGTGTGGAAAGTCCCCAGGCTCCCCAGCAGGCAGAAGTATGCAAAGCATGCATCTCAATTAGTCAGCAACCAgGATCC

SK + HAROSACAGGFPpASVDTSsgROSA:

gatctGGTGTGGAAAGTCCCCAGGCTCCCCAGCAGGCAGAAGTATGCAAAGCATGCATCTCAATTAGTCAGCAACCAGTGGTGTATGAAACTAATCTGTCTGGgGATC

the partial SV40 DTS (72-bp enhancer region),

2. 72-bp-repeat SV40

5′ GGTGTGGAAAGTCCCCAGGCTCCCCAGCAGGCAGAAGTATGCAAGCATGCATCTCAATTAGTCAGCAACCA-3′

#### Long ssDNA donor

DNA templates containing T7 promoter (linearized plasmid or PCR product) were first *in vitro*-transcribed using T7 Ultra mMessage mMachine Kit (Ambion). These IVT RNAs were reverse-transcribed with SuperScript III RT (ThermoFisher) to generate an hybrid RNA-DNA which was treated by RNase H (ThermoFisher) in order to remove RNA from the hybrid molecule and generate ssDNA. A purification step was added to clean the ssDNA with Nucleospin Gel and PCR clean-up (Macherey-Nagel)^20^.

#### OVL strategy

In order to increase the size of the ssDNA used for knockin, we built an overlapping series of ssODNs. The structure of the first ssODN consists of 33 nt 5′HA *rEphX2* followed by 87 nt of GFP sequence. The second ssODN is made of the last 33 nt of the previous ssODN followed by 87 nt of GFP sequence. The third ssODN is made of the last 33 nt of the previous ssODN followed by 54 nt of the GFP sequence and 33 nt 3′HA *rEphX2* ^21^.

#### Donor DNA CX-Rhodamin labeling

For confocal tracking of DNA donor in embryos after electroporation, we used the DNA labeling IT® kit (Mirus) with 8 µg of linear dsDNA CAG-GFP donor.

### Collect, electroporation and microinjection of rat one-cell embryos

Prepubescent females (4–5 weeks old) were super-ovulated with pregnant mare serum gonadotropin (30 IU; Intervet, France) and followed 48 hours later with human chorionic gonadotropin (20 IU; Intervet, France) before breeding. Fertilized 1-cell stage embryos were collected and kept in M16 medium at 37 °C under 5% CO2 until electroporation.

sgRNA and Cas9 protein were incubated at room temperature for 10 min to allow formation of ribonucleoprotein complexes. For KI experiments, DNA template was added to sgRNA/Cas9 protein complexes. The mixture was kept on ice until electroporation or microinjection.

Electroporation was performed using the NEPA21 system (NEPA GENE Co. Ltd, Sonidel, Ireland), a 3-step pulses system: the first one with high voltage and short duration (“poring pulse”) to make micro-holes in the ZP and cytoplasmic membrane, and the second and the third ones with low voltage and long duration (“transfer pulses”) to transfer DNA, mRNA, protein cargos into zygotes cytoplasm. The polarity change of the second transfer pulses increases the transfer efficiency. Briefly, 30–50 zygotes were washed in PBS and loaded in line between the two parallel 5 mm gap platinum plate electrodes on the glass Petri dish (CUY520P5, NEPA GENE Co. Ltd) (Fig. S1), connected to the electroporator. The chamber was filled with 50 µl PBS containing sgRNA/Cas9/DNA at various concentrations. Different poring pulses parameters were tested (voltage: 250 V or 300 V; pulse width: 0.5 or 2.5 ms; number of pulses: 4; pulse interval: 50 ms) to optimize the method. The first and second transfer pulses were unchanged (voltage: 20 V; pulse width: 50 ms; number of pulses: 5; pulse interval: 50 ms).

Microinjection of the ribonucleic complexes with or without donor DNA template was performed into the male pronucleus using an inverted microscope (Nikon Corporation, Tokyo, Japan) and micromanipulators (Narishige, Tokyo, Japan).

Electroporated or microinjected embryos were maintained under 5% CO_2_ at 37 °C until implantation. Surviving embryos were implanted the same day in the oviduct of pseudo-pregnant females (0.5dpc) and allowed to develop until embryonic day 14 or until birth.

### Genotyping

Briefly, E14 or tail biopsies (w6 mm) from 8- to 10-day-old rats were digested overnight at 56 °C, in 1 mL or 500 µL, respectively, of tissue digestion buffer (Tris-HCl 0.1 mol/L pH 8.3, EDTA 5 mmol/L, SDS 0.2%, NaCl 0.2 mol/L, PK 100 µg/mL ). The CRISPRs nuclease targeted regions were PCR amplified from diluted lysed samples (1/20) with a high-fidelity polymerase (Herculase II fusion polymerase). To detect gene edition, locus specific primers *Rosa26*, *Ephx2* or *F* *lna* (Supplementary Table S1) are used and mutations were analyzed by HMA-CE and direct sequencing of PCR products. *Ephx2* and *FlnA* ssODN insertion introduces a *XbaI* or *AatII* restriction site, respectively, in the PCR product, which allows easy detection of KI animals by simple digestion. For *Rosa26* locus, animals were exposed to UV lamp to detect GFP insertion and confirmed by GFP PCR amplification and by amplification with primers located outside and inside of each extremity of the homology arms on this locus (Supplementary Table S1).

### Confocal microscope analyses

Electroporated zygotes were culture overnight and observed using a confocal microscope (Nikon A1RSi, Minato-ku, Tokyo, Japan; oil-immersion objective Plan Apo 60X, NA = 1.4). A 543 nm laser was used to excite the rhodamine 123 fluorophore. Localization of labeled DNA was analyzed for multiple Z-stacks in each zygote. The images were recorded with NIS Element software (Version 4.2, Nikon, Tokyo, Japan).

### Ethics approval

All the animal care and procedures performed in this study were approved by the Animal Experimentation Ethics Committee of the Pays de la Loire region, France, in accordance with the guidelines from the French National Research Council for the Care and Use of Laboratory Animals (Permit Numbers: CEEA-PdL-2015-692).

### Availability of data and materials

The datasets generated and/or analyzed during the current study are available from the corresponding author on reasonable request.

All data generated or analyzed during this study are included in this published article [and its supplementary information files].

## Electronic supplementary material

Supplementary information
