# Supplementary material for: Generation of gene-edited rats by delivery of CRISPR/Cas9 protein and donor DNA into intact zygotes using electroporation
Source: Sci Rep. 2017 Nov 29;7:16554. doi: 10.1038/s41598-017-16328-y (PMC5707420; doi:10.1038/s41598-017-16328-y)
Supplement: Supplementary file 1 — Supplementary information [file 41598_2017_16328_MOESM1_ESM.doc]

**Supplementary information**

Generation of gene-edited rats by delivery of CRISPR/Cas9 protein and donor DNA into intact zygotes using electroporation.

|  |
| --- |

Séverine Remy1,2,3*§, Vanessa Chenouard1,2,3*, Laurent Tesson1,2,3, Claire Usal1,2,3, Séverine Menoret1,2,3, Lucas Brusselle1,2,3, Jean-Marie Heslan1,2,3,4, Tuan Huan Nguyen1,2,4, Jeremy Bellien5, Jean Merot6, , Anne de Cian7, Carine Giovannangeli7, Jean-Paul Concordet7 et Ignacio Anegon1,2,3§.

1Centre de Recherche en Transplantation et Immunologie UMR1064, INSERM, Université de Nantes, Nantes, France.

2Institut de Transplantation Urologie Néphrologie (ITUN), CHU Nantes, Nantes, France.

3Platform Transgenic Rats and ImmunoPhenomics, INSERM UMR 1064-CRTI, F44093 Nantes, France.

4Platform GenoCellEdit, INSERM UMR 1064-CRTI, F44093 Nantes, France.

5INSERM U1096, F76031 Rouen, France.

6Institut du thorax, INSERM UMR 1087, CNRS UMR 6291, F44007 Nantes, France.

7INSERM U565, CNRS UMR7196, Museum National d’Histoire Naturelle, F75005 Paris, France.

*equal contribution

**Supplementary Figure S1**


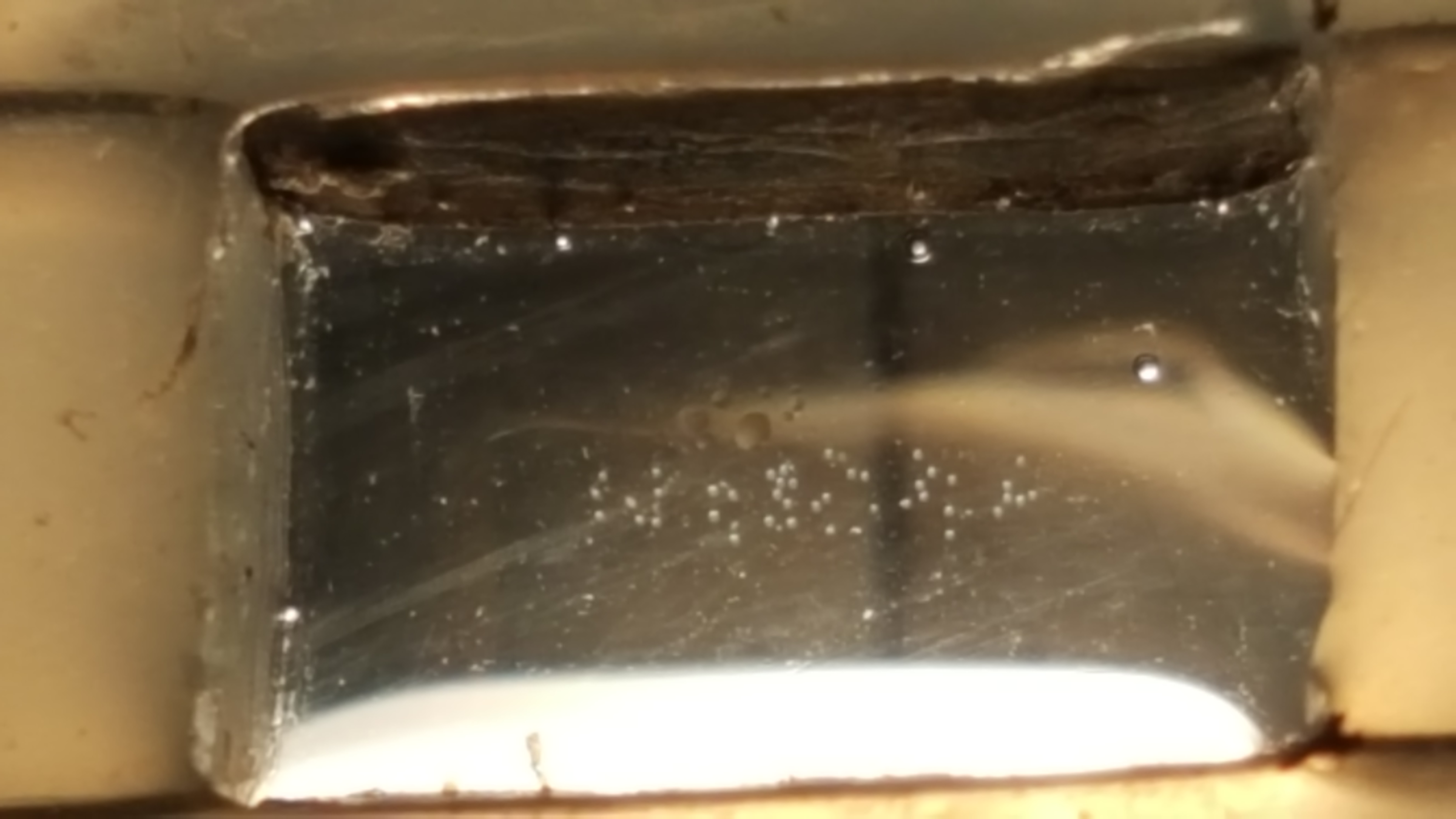

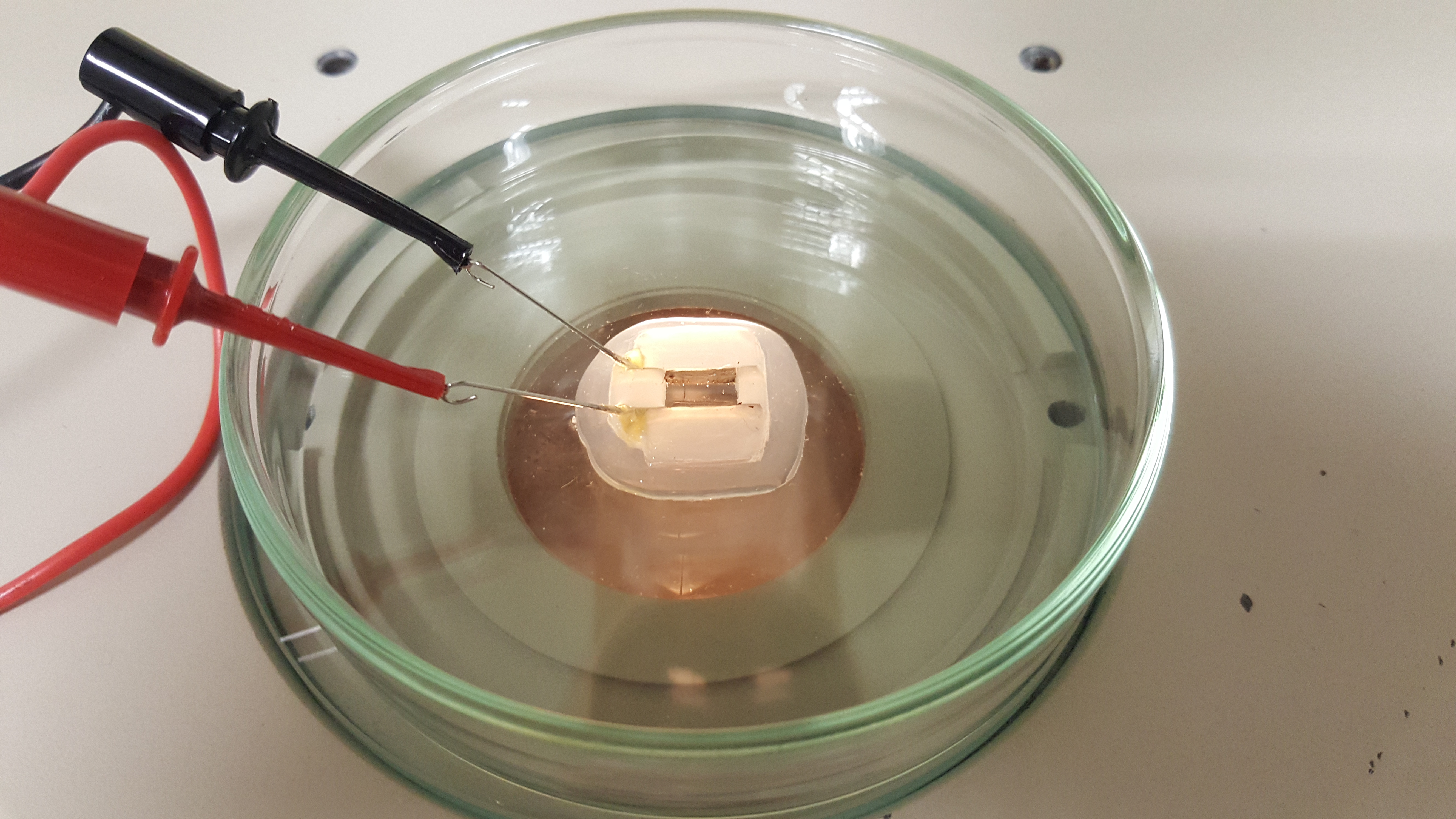


Zygotes are loaded in line between the two parallel 5mm gap platinum plate electrodes on the glass Petri dish (CUY520P5, NEPA GENE Co. Ltd) connected to the electroporator. The chamber was filled with 50µl PBS containing sgRNA/Cas9/DNA at various concentrations.

**a**

**Voltage (V)**

**Pulse width (ms)**

**250**

**0.5**

**250**

**2.5**

**300**

**0.5**

**300**

**2.5**

***

***

***

**

*

**Supplementary Figure S2**

**Impact of voltage and pulse width on egg viability and embryo development**

**b**

**Voltage (V)**

**Pulse width (ms)**

**250**

**0.5**

**250**

**2.5**

**300**

**0.5**

**300**

**2.5**

**

***

**c**

**Voltage (V)**

**Pulse width (ms)**

**250**

**0.5**

**250**

**2.5**

**300**

**0.5**

**300**

**2.5**

***

***

**

***

Survival rate of electroporated embryo 1h after electroporation **(a)**, after one night in culture **(b)** and 14 days post-reimplantation **(c)**. Poring pulses with a voltage of 250V or 300V and duration of 0.5ms to 2.5ms were applied. Data obtained after scoring all loci and all donor DNA/CRISPR-Cas9 conditions. * p<0.05 (Newman-Keuls comparison test)

**Supplementary Table S1.**

Primer sequences

| **Primer name** | **5’-3’ Sequence** |
| --- | --- |
| NHEJ detection   | rROSAfw1  rROSArev1  rEPHX2fw  rEPHX2rev  rFlnA Up  rFlnA Lo |  | | --- | --- | | TGAACTGTGAATAGGCCCAAGTG  GCATTTTAAAAGAGCCCAGTACTTCA  ggcagggtttctagttcttgg  tcttgtaaactgaggcgggta  GTAGAGGCCATTGGTGATGATGT  CAAGGTAAGGCCACCAAAGTC |
| ***Donor insertion***  GFP Up  GFP Lo3 | CCTCGTGACCACCCTGACCT  TCCATGCCGAGAGTGATCCC |
| ***In/out PCR***  rROSA26-5outFor  rROSA26-3outRev  5CAGpRev  3BGHpA-Up2 | TCCCACCCTCCCCTTCCTCT  TGGGTATCACTGGCTGTCCTAGATA  GGCTATGAACTAATGACCCCGTAAT  CCAGATTTTTCCTCCTCTCCTG |

Primers are listed for NHEJ detection, donor insertion, and in-out PCR

**Supplementary Table S2.**

Sequence analyses of the clones PCR products from *Ephx2* positive embryos.

| **Embryo ID** | **Genotype** | **Sequence Forward** | **No. of clones** |
| --- | --- | --- | --- |
| #c | Ins1 (T) | TGCGTGTGGCCGCGTTCGACCTT**T**GACGGAGTGC | 8/11 |
|  | *XbaI* knock-in | TGCGTGTGGCCGCGTTCG**ct**CT**a**GACGGAGTGC | 3/11 |
| #d | WT | TGCGTGTGGCCGCGTTCGACCTTGACGGAGTGC | 7/8 |
|  | Δ10 (GTGGCCGCGT) + *XbaI* knock-in | TGCGT----------TCG**ct**CT**a**GACGGAGTGC | 1/8 |
| #e | WT | visible on F0 sequence | 0/6 |
|  | Δ311 | visible on F0 sequence | 0/6 |
|  | Δ6 (CTTGAC) | TGCGTGTGGCCGCGTTCGAC------GGAGTGC | 3/6 |
|  | *XbaI* knock-in | TGCGTGTGGCCGCGTTCG**ct**CT**a**GACGGAGTGC | 3/6 |

Ins, insertion

Δ, deletion

XbaI site is underlined and small letters indicating mutations introduced in the genome

Survival rate of electroporated embryo 1h after electroporation (**A**), after one night in culture (**B**) and 14 days post-reimplantation (**C**). Poring pulses with a voltage of 250V or 300V and duration of 0.5ms to 2.5ms were applied. Data obtained after scoring all loci and all donor DNA/CRISPR-Cas9 conditions. * p<0.05 (Newman-Keuls comparison test)
